# Supplementary material for: A Likelihood Approach for Real-Time Calibration of Stochastic Compartmental Epidemic Models
Source: PLoS Comput Biol. 2017 Jan 17;13(1):e1005257. doi: 10.1371/journal.pcbi.1005257 (PMC5240920; doi:10.1371/journal.pcbi.1005257)
Supplement: S1 File — (TAR.GZ) [file pcbi.1005257.s014.tar.gz › HSPH_Online-SI-Revision/Additions/PF09/n100-extreme_noisy/n100-extreme_noisy_PF2_table-8.pdf]

1	newly 3 weeks spec	1833.	906.97	{653., 1257.}	50.52	0.320089	newly 3 weeks cumul	3045.73	2065.44	{1560., 2736.}	32.2791	0.180066	newly 5 weeks spec	5869.58	1581.89	{1042., 2449.}	73.0494	0.592729	newly 5 weeks cumul	12 428.	4845.5	{3467., 6927.}	61.0113	0.425436	newly 62 weeks cumul	94 760.2	75 331.4	{70 509., 81 802.}	20.5031	0.100426
2	newly 3 weeks spec	2838.	1236.61	{954., 1691.}	56.4267	0.369979	newly 3 weeks cumul	5925.	2894.37	{2229., 3955.}	51.1499	0.319305	newly 5 weeks spec	5914.	2014.81	{1475., 2775.}	65.9315	0.478167	newly 5 weeks cumul	16 050.	6514.15	{5049., 8875.}	59.4134	0.400693	newly 58 weeks cumul	82 551.	73 427.7	{66 051., 77 002.}	11.0517	0.0519237
3	newly 3 weeks spec	1557.	459.59	{189., 659.}	70.9204	0.59141	newly 3 weeks cumul	2607.	1002.32	{439., 1448.}	62.5631	0.467398	newly 5 weeks spec	6571.	925.53	{304., 1439.}	85.9149	0.943357	newly 5 weeks cumul	12 454.	2577.18	{993., 3892.}	79.3064	0.752746	newly 63 weeks cumul	90 095.	74 027.9	{65 146., 83 409.}	17.9568	0.0883108
4	newly 3 weeks spec	302.	800.82	{90., 1538.}	220.709	0.381813	newly 3 weeks cumul	421.	1306.63	{199., 2592.}	238.52	0.339439	newly 5 weeks spec	3492.	2354.61	{160., 7061.}	103.162	0.823368	newly 5 weeks cumul	5026.	5193.64	{485., 13 085.}	119.571	0.625812	newly 67 weeks cumul	90 300.	75 265.8	{51 358., 93 025.}	17.9918	0.0963399
5	newly 3 weeks spec	841.	378.27	{136., 768.}	58.4863	0.439702	newly 3 weeks cumul	1316.	817.95	{327., 1531.}	47.6299	0.303375	newly 5 weeks spec	3644.	803.61	{215., 1864.}	78.829	0.788609	newly 5 weeks cumul	6776.	2179.97	{726., 4377.}	69.3174	0.591885	newly 64 weeks cumul	88 461.	72 586.	{56 466., 86 198.}	18.4426	0.0929656
6	newly 3 weeks spec	1468.	447.93	{230., 851.}	69.4871	0.570276	newly 3 weeks cumul	2440.	1009.24	{537., 1815.}	58.6377	0.42986	newly 5 weeks spec	5118.	828.44	{393., 1665.}	83.8132	0.866003	newly 5 weeks cumul	10 351.	2452.87	{1224., 4649.}	76.3031	0.684702	newly 62 weeks cumul	91 404.	73 328.8	{66 094., 81 863.}	19.775	0.0973487
7	newly 3 weeks spec	1624.	467.53	{364., 570.}	71.2112	0.546592	newly 3 weeks cumul	2704.	1095.51	{896., 1333.}	59.4856	0.397805	newly 5 weeks spec	5875.	790.04	{630., 973.}	86.5525	0.878514	newly 5 weeks cumul	11 979.	2495.71	{2003., 3074.}	79.166	0.68723	newly 60 weeks cumul	90 884.	73 272.6	{71 242., 75 462.}	19.3779	0.0938571
8	newly 3 weeks spec	1359.	482.41	{255., 762.}	64.5026	0.487402	newly 3 weeks cumul	2461.	1090.15	{606., 1645.}	55.703	0.384837	newly 5 weeks spec	5170.	879.66	{426., 1462.}	82.9853	0.825332	newly 5 weeks cumul	10 407.	2624.36	{1352., 4296.}	74.7827	0.640692	newly 62 weeks cumul	92 361.	72 770.6	{63 034., 80 987.}	21.2107	0.10529
9	newly 3 weeks spec	953.	348.88	{203., 611.}	63.5782	0.47461	newly 3 weeks cumul	1486.	805.14	{502., 1317.}	47.8937	0.305131	newly 5 weeks spec	4055.	614.9	{329., 1148.}	84.836	0.871839	newly 5 weeks cumul	7477.	1890.4	{1096., 3327.}	74.7171	0.637663	newly 62 weeks cumul	92 411.	72 276.3	{66 477., 79 609.}	21.7883	0.107599
10	newly 3 weeks spec	629.	388.44	{67., 763.}	60.3021	0.452078	newly 3 weeks cumul	952.	752.62	{180., 1370.}	55.166	0.33981	newly 5 weeks spec	4393.	1134.04	{140., 2455.}	81.0981	0.916259	newly 5 weeks cumul	6990.	2561.99	{435., 5171.}	72.7069	0.685668	newly 66 weeks cumul	75 317.	71 423.7	{43 449., 92 352.}	19.7716	0.0969272
11	newly 3 weeks spec	2514.	1146.82	{698., 1661.}	54.3827	0.361844	newly 3 weeks cumul	4845.	2570.6	{1625., 3524.}	46.9432	0.292428	newly 5 weeks spec	6044.	2062.34	{1153., 3168.}	65.8779	0.495543	newly 5 weeks cumul	15 023.	6172.23	{3694., 8962.}	58.9148	0.408625	newly 60 weeks cumul	84 746.	75 731.4	{64 168., 84 152.}	10.7077	0.0509853
12	newly 3 weeks spec	1011.	386.42	{303., 456.}	61.7784	0.424346	newly 3 weeks cumul	1634.	898.68	{727., 1048.}	45.0379	0.265127	newly 5 weeks spec	4508.	656.43	{505., 803.}	85.4386	0.84549	newly 5 weeks cumul	8400.	2061.3	{1635., 2444.}	75.4607	0.616481	newly 61 weeks cumul	92 398.	73 614.3	{71 201., 75 690.}	20.3291	0.0989438
13	newly 3 weeks spec	2062.	729.22	{595., 919.}	64.6353	0.457733	newly 3 weeks cumul	3828.	1699.76	{1404., 2080.}	55.5967	0.358137	newly 5 weeks spec	4926.	1221.19	{948., 1537.}	75.2093	0.613242	newly 5 weeks cumul	12 079.	3868.17	{3147., 4839.}	67.9761	0.500799	newly 59 weeks cumul	90 371.	74 161.1	{69 688., 76 765.}	17.9371	0.0861071
14	newly 3 weeks spec	207.	329.26	{106., 716.}	85.2657	0.251767	newly 3 weeks cumul	282.	711.13	{261., 1391.}	156.188	0.339536	newly 5 weeks spec	1855.	695.9	{170., 1858.}	69.0135	0.619595	newly 5 weeks cumul	2833.	1891.01	{580., 4615.}	59.2414	0.376971	newly 64 weeks cumul	71 218.	69 837.6	{51 531., 89 926.}	13.4792	0.0605394
15	newly 3 weeks spec	874.	387.91	{112., 783.}	62.8158	0.488855	newly 3 weeks cumul	1434.	816.95	{278., 1585.}	54.6325	0.366658	newly 5 weeks spec	4922.	857.37	{183., 2198.}	82.7568	0.941437	newly 5 weeks cumul	8480.	2257.27	{603., 5114.}	74.8407	0.711756	newly 64 weeks cumul	79 509.	72 215.3	{57 996., 90 513.}	13.9501	0.0669073
16	newly 3 weeks spec	582.	335.36	{103., 770.}	57.9622	0.398502	newly 3 weeks cumul	805.	718.89	{250., 1448.}	46.5354	0.236447	newly 5 weeks spec	4823.	721.86	{153., 1929.}	85.033	0.991682	newly 5 weeks cumul	7337.	1939.82	{524., 4662.}	74.3187	0.702884	newly 64 weeks cumul	91 266.	72 402.2	{58 712., 89 828.}	20.7247	0.104946
17	newly 3 weeks spec	2320.	702.63	{579., 851.}	69.7142	0.523765	newly 3 weeks cumul	4411.	1654.4	{1383., 1955.}	62.4938	0.430111	newly 5 weeks spec	5419.	1177.6	{956., 1448.}	78.2691	0.668845	newly 5 weeks cumul	13 371.	3749.44	{3164., 4549.}	71.9584	0.556921	newly 59 weeks cumul	87 876.	73 777.6	{70 096., 76 252.}	16.0435	0.0763584
18	newly 3 weeks spec	1347.	483.9	{366., 621.}	64.0757	0.452965	newly 3 weeks cumul	2379.	1133.74	{861., 1369.}	52.3438	0.329232	newly 5 weeks spec	4153.	824.41	{604., 1063.}	80.149	0.711477	newly 5 weeks cumul	9012.	2587.99	{1917., 3217.}	71.2828	0.549999	newly 60 weeks cumul	79 943.	73 505.1	{71 506., 76 419.}	8.05316	0.0369893
19	newly 3 weeks spec	927.	335.6	{206., 631.}	63.9417	0.482629	newly 3 weeks cumul	1494.	770.3	{498., 1395.}	50.7979	0.330822	newly 5 weeks spec	4254.	598.09	{343., 1221.}	85.9405	0.910699	newly 5 weeks cumul	7918.	1817.77	{1126., 3382.}	77.0426	0.683802	newly 62 weeks cumul	93 229.	72 560.2	{67 395., 79 932.}	22.17	0.109905
20	newly 3 weeks spec	53.	476.68	{65., 1001.}	803.774	0.70359	newly 3 weeks cumul	74.	853.82	{153., 1863.}	1055.	0.855615	newly 5 weeks spec	1059.	1550.01	{101., 3694.}	134.773	0.544452	newly 5 weeks cumul	1395.	3318.54	{355., 7095.}	188.37	0.414419	newly 67 weeks cumul	91 568.	71 635.5	{47 376., 92 492.}	22.1759	0.122677
21	newly 3 weeks spec	845.	368.25	{139., 754.}	57.7053	0.44735	newly 3 weeks cumul	1345.	784.91	{319., 1531.}	47.7688	0.319858	newly 5 weeks spec	4282.	812.05	{215., 2064.}	81.0357	0.86759	newly 5 weeks cumul	7677.	2141.47	{699., 4904.}	72.1054	0.655793	newly 64 weeks cumul	83 732.	71 478.2	{55 389., 90 042.}	17.7225	0.0884258
22	newly 3 weeks spec	804.	327.82	{102., 634.}	60.8234	0.488668	newly 3 weeks cumul	1237.	687.03	{246., 1247.}	49.4931	0.348009	newly 5 weeks spec	4322.	768.51	{167., 1591.}	82.2186	0.912283	newly 5 weeks cumul	7480.	1959.61	{519., 3796.}	74.002	0.698692	newly 65 weeks cumul	84 795.	73 475.1	{57 576., 90 157.}	16.0567	0.0793272
23	newly 3 weeks spec	2606.	878.62	{684., 1036.}	66.2847	0.478278	newly 3 weeks cumul	5006.	2072.94	{1621., 2469.}	58.5909	0.38821	newly 5 weeks spec	5905.	1424.07	{1120., 1723.}	75.8837	0.624758	newly 5 weeks cumul	15 055.	4619.41	{3659., 5473.}	69.3164	0.519064	newly 58 weeks cumul	84 036.	73 256.1	{70 810., 75 198.}	12.8277	0.0597595
24	newly 3 weeks spec	1803.	701.76	{610., 827.}	61.0782	0.412946	newly 3 weeks cumul	3172.	1652.57	{1468., 1926.}	47.9013	0.285578	newly 5 weeks spec	5220.	1136.72	{980., 1315.}	78.2238	0.665124	newly 5 weeks cumul	11 528.	3688.42	{3241., 4215.}	68.0047	0.497424	newly 58 weeks cumul	92 404.	72 806.4	{71 564., 74 270.}	21.2086	0.103575
25	newly 3 weeks spec	784.	373.2	{277., 472.}	52.398	0.332646	newly 3 weeks cumul	1188.	862.95	{643., 1080.}	28.4251	0.151652	newly 5 weeks spec	4655.	636.49	{453., 853.}	86.3267	0.875755	newly 5 weeks cumul	7743.	1982.74	{1453., 2541.}	74.3931	0.601166	newly 61 weeks cumul	89 551.	73 751.6	{70 836., 76 167.}	17.643	0.0844941
26	newly 3 weeks spec	2337.	764.39	{610., 911.}	67.2918	0.491985	newly 3 weeks cumul	4561.	1788.47	{1458., 2124.}	60.7878	0.412546	newly 5 weeks spec	5505.	1277.49	{1025., 1561.}	76.794	0.642789	newly 5 weeks cumul	13 758.	4057.73	{3252., 4860.}	70.5064	0.537152	newly 59 weeks cumul	89 914.	73 816.7	{69 485., 76 291.}	17.903	0.0860222
27	newly 3 weeks spec	3178.	1445.88	{995., 2007.}	54.5035	0.356985	newly 3 weeks cumul	6240.	3211.24	{2331., 4239.}	48.5378	0.300443	newly 5 weeks spec	6900.	2593.04	{1602., 3665.}	62.4197	0.4461	newly 5 weeks cumul	18 218.	7777.69	{5293., 10 677.}	57.3077	0.385862	newly 60 weeks cumul	90 939.	76 373.4	{67 356., 82 370.}	16.0169	0.0771171
28	newly 3 weeks spec	1642.	563.24	{218., 991.}	66.5189	0.557013	newly 3 weeks cumul	2833.	1146.92	{526., 1960.}	60.6516	0.467004	newly 5 weeks spec	6075.	1332.77	{359., 2466.}	78.0614	0.80764	newly 5 weeks cumul	12 201.	3360.23	{1193., 5987.}	72.6109	0.669056	newly 64 weeks cumul	93 780.	75 154.1	{58 404., 90 758.}	19.879	0.102457
29	newly 3 weeks spec	2186.	849.45	{672., 1010.}	61.1414	0.415271	newly 3 weeks cumul	4260.	2008.04	{1607., 2347.}	52.8629	0.330954	newly 5 weeks spec	5160.	1388.8	{1110., 1716.}	73.0853	0.576116	newly 5 weeks cumul	12 858.	4490.66	{3592., 5387.}	65.075	0.461794	newly 58 weeks cumul	82 934.	73 276.7	{69 857., 75 449.}	11.6446	0.0539849
30	newly 3 weeks spec	404.	696.59	{79., 1335.}	151.601	0.417515	newly 3 weeks cumul	607.	1161.4	{188., 2345.}	143.911	0.31906	newly 5 weeks spec	3670.	1890.02	{138., 5545.}	94.4774	0.864784	newly 5 weeks cumul	5636.	4324.4	{435., 11 393.}	103.2	0.666575	newly 67 weeks cumul	93 251.	72 217.6	{47 712., 92 937.}	22.6758	0.124531
31	newly 3 weeks spec	169.	541.01	{14., 1184.}	278.574	0.636438	newly 3 weeks cumul	246.	895.9	{32., 1949.}	302.309	0.611385	newly 5 weeks spec	2181.	1904.83	{20., 5827.}	119.785	1.05214	newly 5 weeks cumul	3075.	3926.84	{66., 10 550.}	139.121	0.839771	newly 68 weeks cumul	88 759.	61 639.2	{23 478., 89 885.}	31.3129	0.436491
32	newly 3 weeks spec	874.	417.87	{149., 711.}	62.365	0.458935	newly 3 weeks cumul	1385.	858.69	{356., 1461.}	52.935	0.334914	newly 5 weeks spec	4137.	1012.9	{236., 1901.}	76.9456	0.788693	newly 5 weeks cumul	7503.	2525.18	{845., 4427.}	70.45	0.61258	newly 65 weeks cumul	77 539.	74 122.5	{55 892., 91 139.}	15.1869	0.0700632
33	newly 3 weeks spec	1347.	457.65	{174., 795.}	67.2316	0.551319	newly 3 weeks cumul	2306.	1005.77	{444., 1622.}	59.2467	0.431761	newly 5 weeks spec	4307.	890.15	{256., 1613.}	79.3325	0.807533	newly 5 weeks cumul	9125.	2541.86	{926., 4401.}	72.4294	0.6445	newly 63 weeks cumul	92 452.	68 320.8	{53 988., 79 867.}	26.1013	0.136962
34	newly 3 weeks spec	2211.	1013.39	{829., 1234.}	54.166	0.34504	newly 3 weeks cumul	4652.	2374.07	{1954., 2807.}	48.9667	0.297343	newly 5 weeks spec	4361.	1636.51	{1235., 2055.}	62.474	0.433267	newly 5 weeks cumul	12 123.	5308.46	{4202., 6458.}	56.2117	0.3647	newly 58 weeks cumul	74 850.	72 976.7	{66 869., 76 066.}	3.46754	0.0161281
35	newly 3 weeks spec	845.	400.27	{197., 636.}	54.4438	0.383863	newly 3 weeks cumul	1377.	883.43	{454., 1332.}	40.4902	0.251053	newly 5 weeks spec	3852.	809.36	{339., 1307.}	78.9886	0.758411	newly 5 weeks cumul	7034.	2266.21	{1057., 3576.}	68.154	0.55316	newly 63 weeks cumul	93 735.	74 882.5	{67 209., 82 382.}	20.1126	0.0994103
36	newly 3 weeks spec	2415.	1009.48	{678., 1475.}	58.1996	0.397368	newly 3 weeks cumul	4659.	2235.81	{1610., 3162.}	52.0109	0.332904	newly 5 weeks spec	5307.	1905.45	{1178., 3061.}	64.0955	0.473692	newly 5 weeks cumul	13 623.	5544.76	{3699., 8455.}	59.2985	0.411217	newly 61 weeks cumul	83 417.	74 813.7	{62 862., 82 594.}	10.3183	0.049758
37	newly 3 weeks spec	569.	344.95	{91., 724.}	63.7381	0.45225	newly 3 weeks cumul	794.	675.79	{205., 1245.}	57.2783	0.315563	newly 5 weeks spec	3995.	994.04	{136., 2497.}	81.8969	0.926049	newly 5 weeks cumul	6432.	2263.71	{466., 5114.}	74.7393	0.698464	newly 66 weeks cumul	88 408.	70 838.8	{45 508., 92 746.}	21.6516	0.119827
38	newly 3 weeks spec	174.	357.41	{87., 804.}	130.971	0.304852	newly 3 weeks cumul	245.	710.37	{224., 1417.}	195.049	0.370588	newly 5 weeks spec	2064.	972.92	{141., 2527.}	75.1163	0.656109	newly 5 weeks cumul	2893.	2281.74	{474., 5359.}	72.3291	0.425562	newly 66 weeks cumul	81 546.	69 669.8	{43 392., 91 475.}	20.9423	0.110614
39	newly 3 weeks spec	1689.	589.97	{436., 699.}	65.0699	0.466273	newly 3 weeks cumul	3181.	1369.22	{1064., 1613.}	56.9563	0.374419	newly 5 weeks spec	4543.	990.16	{712., 1198.}	78.2047	0.673157	newly 5 weeks cumul	10 645.	3126.45	{2363., 3730.}	70.6299	0.541546	newly 60 weeks cumul	70 294.	73 656.2	{69 845., 76 829.}	5.9623	0.0251474
40	newly 3 weeks spec	2733.	1133.95	{711., 1610.}	59.1895	0.413429	newly 3 weeks cumul	5234.	2478.13	{1637., 3415.}	53.3158	0.350083	newly 5 weeks spec	6145.	2125.07	{1180., 3284.}	65.7512	0.503122	newly 5 weeks cumul	15 669.	6174.9	{3723., 9082.}	61.0852	0.437574	newly 61 weeks cumul	84 270.	74 192.7	{60 753., 82 985.}	12.128	0.0589626
41	newly 3 weeks spec	2085.	896.13	{482., 1298.}	59.3866	0.425898	newly 3 weeks cumul	3973.	1937.61	{1072., 2717.}	53.8779	0.361133	newly 5 weeks spec	5334.	1770.84	{859., 2680.}	67.85	0.556341	newly 5 weeks cumul	12 888.	4984.66	{2539., 7345.}	62.8982	0.474367	newly 62 weeks cumul	72 211.	74 049.9	{62 608., 83 337.}	10.288	0.0438441
42	newly 3 weeks spec	641.	449.4	{95., 823.}	68.259	0.450723	newly 3 weeks cumul	992.	849.52	{228., 1508.}	63.9778	0.347958	newly 5 weeks spec	4341.	1383.37	{163., 2801.}	81.4303	0.864943	newly 5 weeks cumul	7191.	3052.21	{545., 5796.}	75.5154	0.667637	newly 66 weeks cumul	73 182.	73 297.2	{46 183., 92 657.}	20.1788	0.0939107
43	newly 3 weeks spec	1358.	549.25	{442., 662.}	59.5545	0.399235	newly 3 weeks cumul	2359.	1295.36	{1045., 1539.}	45.0886	0.265428	newly 5 weeks spec	4809.	909.08	{694., 1114.}	81.0963	0.730668	newly 5 weeks cumul	9757.	2913.38	{2261., 3506.}	70.1406	0.530893	newly 59 weeks cumul	92 457.	72 623.7	{70 568., 74 800.}	21.4514	0.105008
44	newly 3 weeks spec	944.	435.37	{153., 646.}	61.7383	0.462341	newly 3 weeks cumul	1464.	871.7	{378., 1245.}	50.6243	0.333693	newly 5 weeks spec	6185.	1083.37	{248., 1661.}	83.9161	0.934983	newly 5 weeks cumul	10 162.	2646.99	{859., 4083.}	76.9027	0.721279	newly 65 weeks cumul	91 937.	76 697.3	{55 244., 90 700.}	16.7573	0.0865079
45	newly 3 weeks spec	599.	345.9	{110., 727.}	53.1786	0.360586	newly 3 weeks cumul	910.	734.76	{276., 1379.}	42.4615	0.23215	newly 5 weeks spec	4808.	743.18	{179., 2054.}	84.5428	0.943108	newly 5 weeks cumul	7429.	1986.86	{603., 4674.}	73.2554	0.668857	newly 64 weeks cumul	82 950.	73 739.3	{61 218., 85 899.}	13.0671	0.0628984
46	newly 3 weeks spec	564.	342.88	{103., 807.}	55.8404	0.378493	newly 3 weeks cumul	898.	738.57	{251., 1607.}	47.2929	0.258689	newly 5 weeks spec	3017.	737.86	{176., 1950.}	75.5433	0.766774	newly 5 weeks cumul	5294.	1990.2	{567., 4807.}	64.0079	0.547721	newly 64 weeks cumul	85 120.	72 351.4	{58 048., 89 962.}	16.617	0.0817194
47	newly 3 weeks spec	765.	370.78	{83., 770.}	65.4248	0.522392	newly 3 weeks cumul	1158.	719.72	{193., 1433.}	56.5475	0.388926	newly 5 weeks spec	3839.	1050.62	{126., 2466.}	80.422	0.871408	newly 5 weeks cumul	6738.	2403.14	{437., 5404.}	73.8293	0.683606	newly 66 weeks cumul	82 631.	71 865.1	{48 098., 92 834.}	18.7212	0.0980885
48	newly 3 weeks spec	268.	376.49	{85., 744.}	91.7201	0.322898	newly 3 weeks cumul	370.	726.59	{189., 1384.}	122.57	0.31355	newly 5 weeks spec	2437.	1097.23	{142., 2520.}	77.1059	0.714268	newly 5 weeks cumul	3631.	2478.35	{452., 5183.}	73.0275	0.498026	newly 66 weeks cumul	91 693.	72 370.2	{47 563., 92 651.}	21.5222	0.11808
49	newly 3 weeks spec	151.	538.89	{22., 1184.}	306.868	0.609407	newly 3 weeks cumul	195.	897.09	{35., 1949.}	390.005	0.641927	newly 5 weeks spec	3032.	1887.6	{24., 5827.}	104.353	1.12905	newly 5 weeks cumul	3868.	3902.2	{95., 10 550.}	120.475	0.868307	newly 68 weeks cumul	88 820.	60 929.4	{15 133., 89 885.}	32.14	0.448222
50	newly 3 weeks spec	2772.	682.61	{552., 829.}	75.3748	0.614233	newly 3 weeks cumul	5085.	1606.9	{1321., 1938.}	68.3992	0.505387	newly 5 weeks spec	6948.	1141.39	{898., 1452.}	83.5724	0.791	newly 5 weeks cumul	16 731.	3633.32	{2981., 4461.}	78.2839	0.668789	newly 59 weeks cumul	91 561.	73 502.8	{69 929., 75 933.}	19.7226	0.0956855
